# Effect of Slow Maxillary Expansion and Alternative Rapid Maxillary Expansion Protocols on Airway Volume in Cleft Palate Cases: A Cone Beam Computed Tomography Based Study

**DOI:** 10.7759/cureus.59534

**Published:** 2024-05-02

**Authors:** Amany M. I. Diab, Basma B. H. Mohammed, Mohamed M Ghoneim, Mohamed A. M. Ali, Sadin Özdemir, Mennat Allah M Shendy, Fehmi Boufahja, Maha M. M. Ali

**Affiliations:** 1 Dental Medicine, Al-Azhar University, Cairo, EGY; 2 Oral and Maxillofacial Surgery, Faculty of Dentistry, Sinai University, El-Arish, EGY; 3 Biology, Imam Mohammad Ibn Saud Islamic University, Riyadh, SAU; 4 Biology, Mersin University, Mersin, TUR; 5 Dentistry, Ministry of Health Holdings, Cairo, EGY; 6 Biology, Imam Muhammad Ibn Saud Islamic University, Riyadh, SAU; 7 Dentistry, Al-Azhar University, Cairo, EGY

**Keywords:** facemask, edo, expansion, alt-ramec, cleft lip and palate (clp), pharyngeal airway volumes

## Abstract

A total of 22 patients with cleft palate aged 8 to 12 years were selected and categorized into two groups: the first group was treated with alternate rapid maxillary expansion and constriction (Alt-RAMEC) using an expander with differential opening (EDO) and facemask, while the second group was treated using slow maxillary expansion (SME) using an EDO. Finally, the pharyngeal airway volume in the two groups was compared using cone beam computed tomography (CBCT). CBCT scans were performed before expansion and six months following the expansion. Alveolar crest level, maxillary breadth, nasal cavity width, arch width, inclination of the molar teeth, buccal and palatal alveolar bone thickness, and maxillary alveolar width were all assessed. Paired t-tests (p=0.05) were applied to compare interphase data. The two groups showed a non-significant difference in terms of nasopharyngeal volume (cm^3^), oropharyngeal volume (cm^3^), and overall pre- and post-treatment results (p>0.005). Results of comparison of pre- and post-treatment periods in the Alt-RAMEC group revealed a significantly higher cleft volume (cm^3^) (p=0.001). Results of comparison of pre- and post-treatment periods in the SME group revealed a substantial rise in cleft volume (cm^3^) (p=0.003). Results from a comparison of the cleft volume (cm^3^) between the two study groups pre- and post-intervention revealed a non-significant difference (p=0.200 and 0.054, respectively).

## Introduction

Cleft lip and palate (CLP) is regarded as a common anomaly in humans, with one per 700 infants affected by this birth deformity [1]. The genesis of this oral deformity is considered multifactorial and may be due to racial, socioeconomic, and geographical factors [2]. Most CLP patients (around 70%) have nasal airway abnormalities, and of those, 80% exhibit varying grades of mouth breathing [3]. Additionally, it has been shown that a smaller nasal airway increases the risk of mouth breathing, sleep apnea, and speech issues including hyponasality [4]. Patients with CLP also complain of soft palate muscular abnormalities that are dysfunctional, which might increase the risk of sleep apnea dyspnea syndrome when combined with maxillary or mandibular retrognathia, a short mandibular body, and a clockwise mandibular rotation [5].

Maxillary constriction in CLP patients was improved using different devices such as Hyrax, Hass, Quid Helix, or fan-type expanders. The essential components of another novel appliance, expander with differential opening (EDO), which works to enlarge both the anterior and posterior portions of the maxillary dental arch, are two parallel-opening screws. Additionally, by addressing the anterior crossbite, maxillary protraction can assist in enhancing the speech issues associated with CLP [6,7].

Luxation of all maxillary sutures without overexpanding the maxillary arch with Alt-RAMEC is one of the explanation protocols that are different from standard rapid or slow protocols [8].

The pharynx of patients with CLP has aberrations due to congenital deficiencies and surgeries such as palatoplasty. With the introduction of cone beam computed tomography (CBCT) and imaging software, it is now possible to generate three-dimensional (3D) images for reliable assessment of airway dimensions [9].

Variable impacts of maxillary expansion and protraction on pharyngeal airway volumes have been discovered in studies. Some studies revealed significant modifications in pharyngeal airway volumes, while other studies found no changes.

An earlier study that evaluated the impact of Alt-RAMEC treatment with a protraction facemask (FM) on pharyngeal airway volume in non-cleft patients used two-dimensional (2D) radiography and discovered a significant increase [10]. Onem Ozbilen et al. reported the same findings as Celikoglu and Buyukcavus, but the main difference is using 3D CBCT radiography [11]. However, after rapid maxillary expansion (RME), oropharyngeal airway volumes of non-cleft patients did not significantly change, according to a retrospective research study by Zhao et al. [12].

Only one prospective clinical study reported the effect of Alt-RAMEC and an FM on cleft patients. Using CBCT and finite element modeling, Singh et al. found significant variations in pharyngeal airway volume [13].

This research aimed to compare the impact of maxillary expansion by EDO using slow maxillary expansion (SME) protocol and FM protraction and the impact of Alt-RAMEC protocol maxillary expansion and FM protraction on pharyngeal airway in patients with CLP using 3D CBCT because there have not been many investigations on airway volume in CLP patients by CBCT.

## Materials and methods

Al-Azhar University Faculty of Dental Medicine for Girls' Research Ethical Committee gave ethical approval for this study, which involved 20 CLP patients (8-12 years old) from the center for treatment at Al-Azhar University and Sayed Galal Hospital in Cairo, Egypt. Before starting therapy, patients' parents were made aware of the study's goals and were then asked to sign an “informed consent” form.

The exclusion criteria included previous orthodontic intervention, trauma, syndromes, history of tonsillectomy or adenoidectomy, treatment record of pertaining positive airway pressure, medication that affects orthodontic tooth movement, such as calcitonin and bisphosphonates, history of upper airway obstruction, record of recurrent colds (more than six times in the previous year), and having a cold or superior airway inflammatory disease throughout the CBCT scan [14].

The following criteria were met by all patients: growing patients with mixed dentition who had their maxillary first permanent molar erupt, lip repair and palatal closure performed previously, no history of orthopedic or orthodontic treatment, and both the parent and the patient being cooperative [15,16].

A total sample size of 20 patients was estimated to be sufficient to detect an effect size of 0.4 at a power of 90% (0.9) at a partial eta squared of 0.14 and at a significance level of 0.05. Sample size was calculated using G*power version 3.1.9.6. Two equal groups of patients were established and given either slow-maxillary expansion (SME) using an EDO or facemask protraction utilizing the Alt-RAMEC procedure (EDO).

Interventions

All patients had maxillary expansion and a protraction petit FM. Orthodontic bands were cemented to maxillary permanent first molars, and bonded acrylic plates were employed, covering all posterior maxillary teeth. Red acrylic was used to mark the palatal cusp tips of the posterior maxillary teeth. The EDO screws, which are placed in the middle of the arch, were soldered to the palatal side of molar bands that were cemented to permanent first molars. On the buccal surface of the first permanent molars, a wire extension with hooks close to the canine region on both sides was soldered for attaching FM elastics. The parents of the patient with the Alt-RAMEC protocol were instructed to activate or deactivate the two expander screws by turning them twice in the morning and evening by two-quarter turns each per day for seven weeks, with a rate of one week of activation followed by one week of deactivation [16].

After seven weeks, patients were directed to wear the small FM for six months while keeping the expander inside the mouth. Extraoral elastics of ORMCO Z-pak elastics (3/8"), 14 Oz, 350 grams per side, were used at 20°-30° angulation downward the occlusal plane. Participants were directed to use an FM for 14-16 hours [11-13].

**Figure 1:**
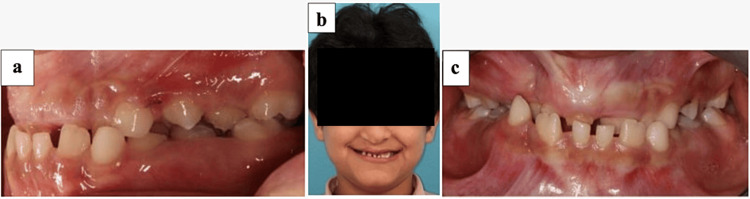
Photographs before treatment. (a) Lateral view. (b) Extraoral frontal view. (c) Profile view.

**Figure 2:**
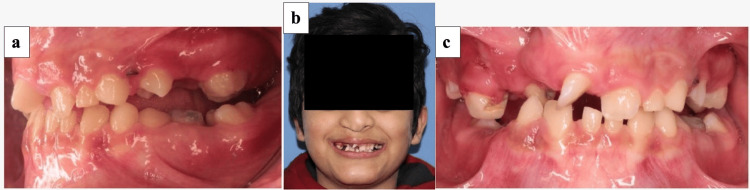
Photographs after expansion. (a) Lateral view. (b) Extraoral frontal view. (c) Profile view.

An SME procedure of one quarter turn every two days (three times weekly) was used to activate the anterior and posterior screws of EDO (Great Lakes Orthodontics, Tonawanda, NY, USA) until a little overcorrection in the canine and molar areas was reached. The buccal cusp tips of three posterior mandibular teeth would be connected by the red acrylic line of EDO. In the molar region, the palatal cusp tips of the posterior maxillary teeth must touch the buccal cusp tips of the posterior mandibular teeth. At the anterior teeth, a slight overcorrection of two millimeters should be achieved in the inter-canine distance. Depending on the degree of maxillary arch constriction, the active phase of expansion lasted anywhere from two to six months. The extent of expander screws was then fastened with ligature wire and remained in the mouth for six months as a retainer. The expander was removed, and a fixed bonded retainer was put in its place at the end of the retention phase. An extended palatal arm rests on the palatal surface of the permanent maxillary premolars/deciduous molars and the anterior teeth, and a trans-palatal arch connects the retainer to the maxillary permanent first molars. Due to the use of an FM appliance to prolong the therapy, the retainer additionally features hooks on both sides in the canine area (Figures 1, 2).

Registration method

**Figure 3:**
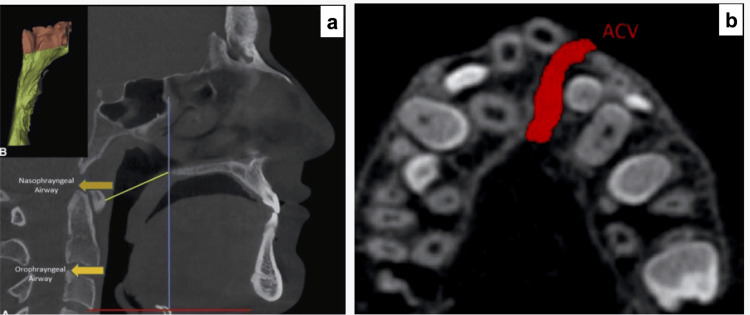
(a) Pharyngeal, oropharyngeal, and nasopharyngeal air volume. (b) Alveolar cleft volume.

**Figure 4:**
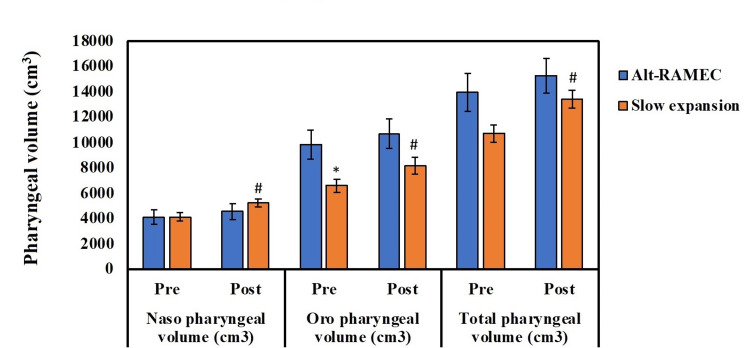
Nasopharyngeal volume, oropharyngeal volume, and total volume of the two study groups. *Significant variation between groups (Alt-RAMEC and SME). #Significant variations between pre- and post-treatment measurements at p ≤ 0.05. Alt-RAMEC, alternative rapid maxillary expansion; SME, slow maxillary expansion; Pre, pre-treatment measurement; Post, post-treatment measurement

For every patient, two CBCT scans were obtained using the Planmeca ProMax 3D Mid-CBCT scanner (Planmeca Oy, Helsinki, Finland). The first scan was conducted immediately before the expansion (T1), and the second scan was conducted from the first to the sixth month after the extension's active phase; the expander was removed to start the retention phase (T2). Scans were stored in DICOM format. The scan parameters were as follows: 90 kVp, 12 mA, 6.2 s of scanning time, 10 cm of field of view, and 0.2 mm of voxel size (Figures 3, 4).

Statistical analyses

Data were handled and explored using SPSS Version 29.0 (IBM Corp, Armonk, NY). Data were checked for normality using the Shapiro-Wilk normality test to check whether the data were parametric or non-parametric. Data were parametric, and, accordingly, average and standard deviation were used to express parametric data. A significance level of 5% was applied. A t-test was used to compare two groups under study with quantitative variables that were regularly distributed. Paired t-tests were applied to analyze the significance between periods.

## Results

Twenty CLP patients, with an average age of 10.57±2.0 years, were divided into two groups. The difference between the two groups in age was non-significant, as revealed by the independent t-test. The first group received SME using an EDO, while the other received EDO and FM protraction with the Alt-RAMEC protocol. Overall, 50% of the population was female and 50% of the population was male.

There were no significant variations between the two groups when the nasopharyngeal volume (cm^3^), oropharyngeal volume (cm^3^), and total pre- and post-treatment measurements were compared among the two intervention groups (p > 0.05); these measurements were taken by two examiner (Table 1, Figure 4).

**Table 1:** Comparison of the nasopharyngeal, oropharyngeal, and total volumes across the two study groups. Data are expressed using mean ± SD. Difference between Alt-RAMEC and SEM was performed by independent t-test presented as p-values. *Significant at p ≤ 0.05 Alt-RAMEC, alternative rapid maxillary expansion; SME, slow maxillary expansion; Pre, pre-treatment measurement; Post, post-treatment measurement; P0, significance between periods using paired t-test

		Alt-RAMEC	SME	P-value
Nasopharyngeal volume (cm^3^)	Pre	4,132.75±1,595.89	4,128.87±889.37	0.995
	Post	4,557.12±1,806.27	5,232.87±807.35	0.350
	P0	0.435	0.006*	
Oropharyngeal volume (cm^3^)	Pre	9,840.50±3,202.91	6,594.25±1,478.36	0.021*
	Post	10,698.25±3,250.11	8,183.37±1,829.69	0.077
	P0	0.543	0.028*	
Total	Pre	13,973.25±4,219.19	10,723.12±1,945.25	0.068
	Post	15,255.37±3,855.34	13,416.25±2,103.16	0.256
	P0	0.479	0.011*	

**Figure 5:**
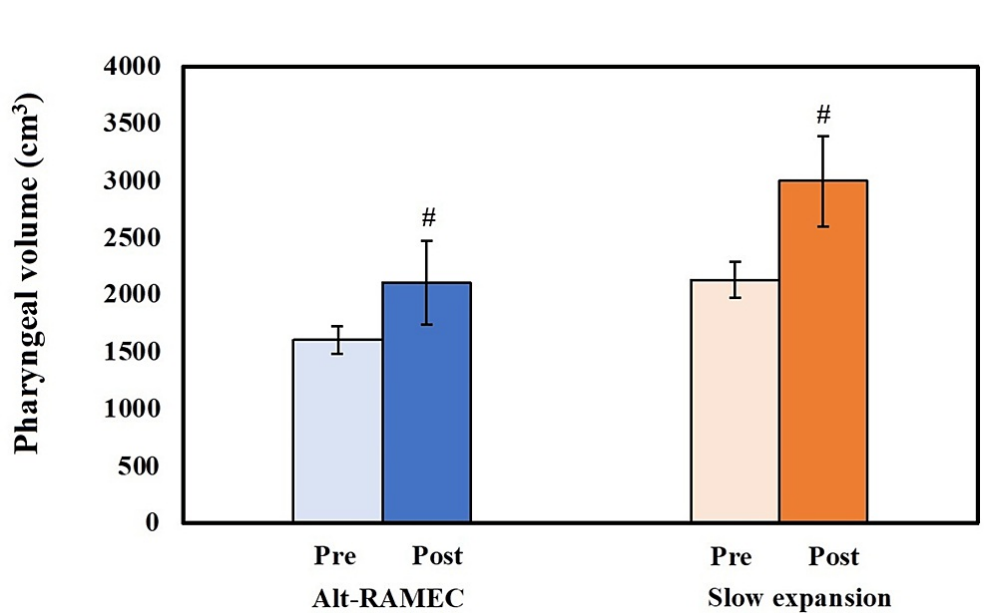
Cleft volume comparison between the two research groups. #Significant different between pre- and post-treatment measurements. Alt-RAMEC, alternative rapid maxillary expansion; Pre, pre-treatment measurement; Post, post-treatment measurement

When the Alt-RAMEC group's pre- and post-treatment data were compared, the cleft volume significantly increased (p=0.001). A substantial increase in cleft volume was observed in the SME group when comparing pre- and post-treatment findings (p=0.003). Comparison between the two studied groups according to cleft volume, pre-treatment and post-treatment, showed a non-significant difference in cleft volume (p=0.200 and 0.054) (Table 2, Figure 5).

**Table 2:** Cleft volume estimation in the two studied groups. *Significant at p ≤ 0.05. Alt-RAMEC, alternative rapid maxillary expansion; SME, slow maxillary expansion; Pre, pre-treatment measurement; Post, post-treatment measurement; P0, significance between periods using paired t-test

	Cleft volume (cm^3^)		P-value
	Alt-RAMEC	SME	
Pre	1,606.50±353.63	2,128.87±1,040.52	0.200
Post	2,105.00±445.95	2,998.25±1,116.84	0.054
P0	0.001*	0.003*	

## Discussion

In this study, non-syndromic CLP patients between 8 and 12 years of age were split into two groups: one group received EDO and FM protraction using the Alt-RAMEC protocol, while the other group received SME using an EDO. We were unable to incorporate a control group into our research since delaying the care of patients with CLP would be unethical.

Protraction of the maxilla may be beneficial because fast palatal growth in people with clefts causes the circummaxillary sutures to disarticulate. These benefits include expanding the arch and aligning it to improve the airway dimensions after expansion, facilitating nasal expansion, and preparing it to assist in maxillary protraction. These are the basic requirements for expansion [17].

Few studies were concerned with the effect of the Alt-RAMEC protocol and FM therapy on pharyngeal airway volume changes. For non-cleft patients, Celikoglu and Buyukcavus [10] used cephalometric 2D radiographs to assess the efficacy of Alt-RAMEC combined with FM treatment on pharyngeal airway volume and used a full-coverage hyrax expander, which revealed a significant increase in upper pharyngeal airway dimensions (p < 0.001), However, it was shown that the alterations in the lower pharyngeal dimension were statistically negligible (p > 0.05).

Even though 2D pictures can also be utilized for this, CBCT scans are preferred since they are more accurate and allow for the determination of airway volume. It is significant as it was shown that breathing difficulties are related to the airway's least cross-section when seen perpendicularly [18]. It should nonetheless be mentioned that CBCT has a characteristic inaccuracy due to the respiratory phase because research suggests that breathing during scanning influences airway size and morphology [19]. However, CBCT is still much more precise than lateral cephalometry for these measures.

Onem Ozbilen et al. [11] used CBCT to estimate and rival modifications in the pharyngeal airway, after RME and Alt-RAMEC, followed by FM and double-hinged expansion screw therapy. He found no significant variations were detected in any of the pharyngeal airway volumes in the RME/FM group. Although the upper pharyngeal airway volume did not substantially rise in the Alt-RAMEC/FM group, the lower and total pharyngeal airway volumes did (1,011.19 and 1,601.21 mm^3^, respectively).

For cleft patients, one prospective clinical study on the use of Alt-RAMEC and FMs is accessible. Singh et al. [13] evaluated pharyngeal airway dimensions using protocols and a custom-made fan-shaped expansion screw. The expansion rate and constriction were 1 mm/day for nine weeks, followed by a six-month FM. A significant variation (p = 0.05) was seen for changes in pharyngeal volume, with an average difference of 1.57.

In both groups, our data showed a significant expansion in oropharyngeal airway volume. However, there was a non-significant variation among both groups when examining airway volume. Therefore, based on our research, there is not a lot of evidence that maxillary expansion and protraction impact the volume of the oropharyngeal airway. In these CPL people, the oropharyngeal airway volume may have increased due to growth rather than expansion and protraction [20]. Children without clefts aged 6 to 20 years showed a constant rise in airway volume, according to Schendel et al. [21]. Kula et al.'s findings, showing that children with CLP exhibit an increase in nasal airway volume due to growth in both unilateral and bilateral CPL, are in line with our own [22].

Our findings were in line with these earlier investigations, which showed that both non-cleft and CPL people saw an increase in pharyngeal airway volume during development. According to Sheng et al. [20], children without clefts had an increase in pharyngeal airway depth over a three- to four-year period, from the stage of mixed dentition to the stage of permanent dentition.

The current findings on the non-significant difference in cleft volume between the two groups were consistent with the findings of a prior study that revealed the lack of a mid-palatal suture as a result of the suture's extremely low or non-existent resistance. Additionally, Alt-RAMEC or slow expanders cause equal expansion in the anterior and posterior regions, which may not be desired in cases with bilateral clefts as the molars are typically in buccal cross-bite and expansion of this type may worsen the issue [17].

The heterogeneous sample group in our study, which included both unilateral and bilateral CLP and cleft palate alone, as well as the uneven gender distribution between the two groups, are its limitations. The effects of protraction and expansion may vary depending on the subject. We were unable to include a control group in our research since delaying patients' intervention for CLP would be unethical. Additionally, in our research, we looked into slow expanders and Alt-RAMEC expanders, both of which have various maxillary expansion designs. Instead of focusing on specific dimensions, this study examined variations in oropharyngeal airway volume, nasopharyngeal airway volume, and overall volume.

## Conclusions

The current research aimed to compare the effects of maxillary expansion by EDO using the SME protocol and FM protraction and the Alt-RAMEC protocol on maxillary expansion and FM protraction in patients with CLP using 3D CBCT. The variations in airway volume and cleft volume among both groups did not show a significant variation, according to our findings. Alt-RAMEC protocol and slow maxillary expansion produced similar airway changes in patients with CLP. EDO expander with both protocols can be used and is useful in the treatment of maxillary constriction in CLP.
